# p53 expression in repair/reactive renal tubular cells: A potential pitfall leading to a false‐positive diagnosis of urine cytology

**DOI:** 10.1002/cam4.4389

**Published:** 2021-11-16

**Authors:** Kaori Enomoto, Toru Matsunaga, Tadashi Sofue, Akihiro Nakamura, Eiichiro Hirakawa, Emi Ibuki, Reiji Haba, Shingo Kamoshida, Hiroyuki Ohsaki

**Affiliations:** ^1^ Department of Medical Biophysics Kobe University Graduate School of Health Sciences Kobe Japan; ^2^ Department of Diagnostic Pathology University Hospital Faculty of Medicine Kagawa University Kagawa Japan; ^3^ Department of Cardiorenal and Cerebrovascular Medicine Faculty of Medicine Kagawa University Kagawa Japan; ^4^ Department of Clinical Laboratory Science Faculty of Health Care Tenri Health Care University Tenri Japan; ^5^ Department of Medical Technology Kagawa Prefectural University of Health Sciences Kagawa Japan

**Keywords:** glomerular disease, immunocytochemistry, p53, renal tubular cell, urine cytology

## Abstract

**Background:**

p53 immunostaining is routinely used as a surrogate marker for *TP53* mutational status. In urine cytology, p53 immunocytochemistry is reportedly useful in detecting urothelial carcinoma cells as well as in improving the detection sensitivity and specificity. However, to the best of our knowledge, p53 expression in repair/reactive renal tubular cells (RRTCs) from urine cytologic specimens has not been assessed to date.

**Methods:**

We evaluated the immunoexpression of p53 and homogentisate 1,2‐dioxygenase (HGD) antibody, a renal tubular cells marker, in RRTCs using voided urine and renal biopsy samples from 80 patients who were histologically diagnosed with glomerular disease.

**Results:**

Repair/reactive renal tubular cells were detected in 68 (68/80, 85%) samples at a mean count of 141.1 cells per sample (range, 5–4220). Immunocytochemical analysis found p53‐positive RRTCs in all the samples (68/68, 100%) with an average p53 positivity rate of RRTCs per sample at 47.7% (range, 3.8%–96.5%). Of the 68 p53‐positive RRTC samples, 38 (55.9%) included cells that were HGD positive for cytoplasm. Similarly, renal biopsy analysis revealed p53‐positive RRTCs in all the specimens (68/68, 100%). All 68 (100%) cases showed RRTCs that were positive for both p53 and HGD.

**Conclusion:**

To avoid false positives of p53 immunocytochemistry, cytologists must consider the fact that RRTCs from patients with glomerular disease are positive for p53.

## Graphical abstract

Although the existence and cytological features of repair/reactive renal tubular cells in urine are not yet widely well known, these cells cytomorphologically mimic cancer cells. This study found that repair/reactive renal tubular cells are positive for p53 immunocytochemistry, which is crucial information for helping to avoid false positives in urine cytology.

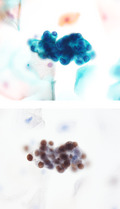

## 1 INTRODUCTION

Urothelial carcinoma is the most common histologic carcinoma type in the urinary tract, including the bladder, renal pelvis, and ureter.^1^, ^2^ *TP53* mutations characterize high‐grade urothelial carcinoma (HGUC) in the dual pathway of urothelial carcinogenesis, with the mutation rate in HGUC being twofold higher than that in low‐grade tumors.^3^ Although the existence of the p53 null phenotype lacking p53 immunoexpression has been recently reported, p53 immunostaining is generally used as a surrogate marker for *TP53* mutational status in urothelial carcinoma.^4^, ^5^ p53 overexpression in the nucleus has been shown not only to correlate with progression and recurrence of urothelial carcinoma but also to be useful in distinguishing urothelial carcinoma in situ from reactive urothelial atypia.^6^, ^7^, ^8^ In urine cytology, p53 immunocytochemistry has been reported as useful in detecting urothelial carcinoma cells (UCCs) as well as in improving their detection sensitivity and specificity.^9^, ^10^, ^11^, ^12^

Repair/reactive renal tubular cells (RRTCs), derived from nephron, frequently appear in urine from patients with glomerular disease.^13^, ^14^ These cells show atypia, including nuclear enlargement, nucleolar prominence, nuclear contour irregularity, and cannibalism.^15^, ^16^ Therefore, because RRTCs mimic UCCs and adenocarcinoma cells, their presence in urine cytologic specimens can lead to overdiagnosis. Although p53 immunostaining has been reported to be positive for UCCs and useful in distinguishing from reactive epithelial cells,^10^, ^11^ renal tubular cells undergoing a morphological change during acute tubular necrosis have also been found to be positive for p53.^17^ In addition, we recently observed p53‐positive RRTCs in renal biopsy and urine cytologic specimens from patients with glomerular disease. p53‐positive RRTCs appearing in urine may lead to a false‐positive diagnosis of urine cytology. However, to the best of our knowledge, no data on p53 immunocytochemistry in RRTCs from urine cytologic specimens have been reported yet.

The objectives of this study were to evaluate p53 immunoexpression in RRTCs using urine cytology and to confirm p53 immunoexpression in the nucleus of RRTCs.

## 2 MATERIALS AND METHODS

### 2.1 Patients and histological diagnosis

This study used voided urine and renal biopsy samples from 80 patients (37 men and 43 women; mean age, 56.8 ± 21.1 years) histologically diagnosed with glomerular disease at the Kagawa University Hospital (Table 1). None of the patients had a history of urinary tract cancer. In this study, since renal tubular damage was particularly important, one of the authors (EI) evaluated the ratio of renal tubular damage in total renal tubular using hematoxylin and eosin‐stained renal biopsy sections.

**TABLE 1:** Histologic diagnosis of glomerular disease in the study population

Histologic diagnosis	*n*
Immunoglobulin A nephropathy	22
Membranous glomerulonephritis	13
Diffuse crescentic glomerulonephritis	7
Obesity‐related glomerulopathy	6
Minor glomerular abnormalities	6
Diabetic glomerulopathy	4
Lupus nephritis	4
Tubulointerstitial nephritis	4
Infection‐related glomerulonephritis	3
Membranoproliferative glomerulonephritis	2
Focal segmental glomerulosclerosis	2
Alport syndrome	1
Amyloidosis	1
Hepatitis C virus‐associated glomerulonephritis	1
Thin membrane disease	1
Fibronectin nephropathy	1
Malignant nephrosclerosis	1
Light chain proximal tubulopathy	1
Total	80

### 2.2 Urine cytology

All urine samples (>30 mL) were obtained just before the renal biopsy and prepared according to a previously described modified version of the SurePath method.^18^ Briefly, urine was centrifuged at 3000 rpm for 2 min, and sediments were resuspended in 10 mL of CytoRich Red (Becton‐Dickinson). The specimen was then centrifuged after a 30‐min fixation. Then, 5 mL of distilled water was added to the sediment, and the specimen was centrifuged again. Another 300 μL of distilled water was added to the sediment for resuspension. The specimen was then transferred into a settling chamber (Becton‐Dickinson) and mounted on a positively charged slide (Becton‐Dickinson) for 15 min. The slide rack (Becton‐Dickinson) was then turned upside down to discard the supernatant, and the inside of the settling chamber was rinsed with 95% alcohol. The settling chamber was removed after the alcohol was discarded, and the slide was immediately placed in fresh 95% alcohol. Subsequently, routine Papanicolaou staining was performed on all SurePath slides.

### 2.3 p53 immunostaining of cytologic and histologic specimens

Papanicolaou‐stained SurePath slides were immersed in xylene at 40℃ to remove the coverslips and residual mounting medium. They were then rehydrated with xylene and an ethanol gradient. Meanwhile, the renal biopsy sections were deparaffinized with xylene and rehydrated with an ethanol gradient. The cytologic and histologic specimens were processed as follows: Endogenous peroxidase activity was blocked with 0.3% hydrogen peroxide in methanol for 10 min. Heat‐induced antigen retrieval using 10 mM citrate buffer (pH 6.0) was performed in a water bath at 90℃ for 20 min for the cytologic specimens and in a pressure cooker for 5 min for the histologic specimens. After antigen retrieval, the specimens were left to rest at room temperature (RT) for 30 min to cool. They were then incubated overnight with anti‐p53 mouse monoclonal antibody (1:200 dilution; clone DO‐7; Dako, Glostrup) at RT. Then, the specimens were incubated for 30 min with anti‐mouse horseradish peroxidase polymer (Histofine Simple Stain MAX‐PO; Nichirei Bioscience) at RT. They were then developed using diaminobenzidine solution (Nichirei Bioscience) and counterstained with Mayer's hematoxylin. We used colorectal cancer histological slides as a positive control for p53 immunostaining of cytologic and histologic specimens. In the present study, in order to prevent the detachment of the cells from the slides at high temperature, antigen retrieval of the cytologic specimens was performed at 90℃.

### 2.4 Homogentisate 1,2‐dioxygenase (HGD) immunostaining of cytologic and histologic specimens

After determining the p53 positivity rate, we performed immunostaining using HGD antibody, a renal tubular cells marker, to confirm the origin of the p53‐positive cells in the urine cytologic specimens. p53‐immunostained cytologic and histologic slides were immersed in xylene at 40℃ to remove the coverslips and residual mounting medium, after which they were rehydrated with xylene and an ethanol gradient. The specimens were then incubated overnight with an anti‐HGD rabbit monoclonal antibody (1:800 dilution) (Abcam) at RT. Then, the specimens were incubated for 30 min with anti‐rabbit alkaline phosphatase polymer (Histofine Simple Stain AP [R]; Nichirei Bioscience) at RT and then developed using new fuchsin solution (Nichirei Bioscience). The slides were washed with tap water and mounted using an aqueous permanent mounting medium (CC/Mount; Diagnostic BioSystems). Based on this double immunostaining theory, p53‐positive RRTCs should contain nuclei that are stained brown and cytoplasms that are stained red. We used normal kidney histological slides as positive control for HGD immunostaining of cytologic and histologic specimens.

### 2.5 Determination of the appearance and positivity rates of RRTCs and categorization according to the Paris system

The appearance rate of RRTCs in each Papanicolaou‐stained urine cytologic specimen was determined under a multiheaded microscope by two authors (KE and HO). The RRTCs were selected based on the following cytomorphological features: hobnail‐shaped cells, intracytoplasmic hemosiderin, vacuolated cytoplasm, prominent nucleoli, a rosette‐like arrangement, and clusters of <50 cells.^14^, ^16^ Concurrently, we examined under which categories these RRTCs belonged to using the Paris system.^19^

The positivity rates of p53‐positive RRTCs and p53/HGD‐positive RRTCs in the urine cytologic and renal biopsy specimens were determined under a light microscope by one of the authors (KE), independently of the strength of the expression.

### 2.6 Statistical analysis

Spearman's rank correlation coefficient was used to assess the correlation between renal tubular damage rate in renal biopsy and the number and p53 positivity rate of urinary RRTCs. The statistical tests were performed with *p* value <0.05. Statistical analysis was performed using the StatFlex software (version 6.0; Artec Inc.).

## 3 RESULTS

### 3.1 Renal tubular damage rate in renal biopsy

A total of 80 renal biopsies from patients with glomerular disease were evaluated using hematoxylin and eosin‐stained renal biopsy sections. Renal tubular damage was detected in 53 (66.3%) cases with a mean count of 17.1% (range, 0%–70%).

### 3.2 Appearance rate and cell count of RRTCs

**FIGURE 1:**
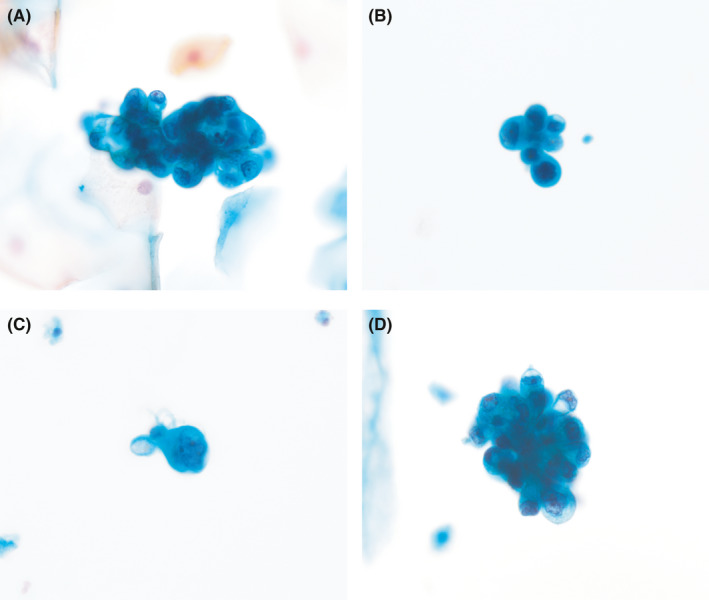
Cytomorphology of repair/reactive renal tubular cells (RRTCs) in urine cytology (Papanicolaou stain). A, Three‐dimensional cluster of RRTCs. B, Small cluster of RRTCs mimicking high‐grade urothelial carcinoma cells. C, Large RRTCs showing a high N/C ratio and irregular nuclear contours. D, Rosette‐like cluster of RRTCs mimicking adenocarcinoma cells

A total of 80 voided urine samples from patients with glomerular disease were cytomorphologically analyzed using Papanicolaou staining. RRTCs were detected in 68 (85.0%) samples at a mean count of 141.1 RRTCs per sample (range, 5–4220; Figure 1).

### 3.3 Categorization of urinary RRTCs according to the Paris system

A total of 68 urinary RRTCs cases were categorized according to the Paris system as follows: 19 cases belonged to negative for HGUC (27.9%), 23 cases to atypical urothelial cells (33.8%), 22 cases to suspicious for HGUC (32.4%), 1 case to HGUC (1.5%), and 3 cases to adenocarcinoma (4.4%).

### 3.4 p53 positivity rate of RRTCs in urine cytology

**FIGURE 2:**
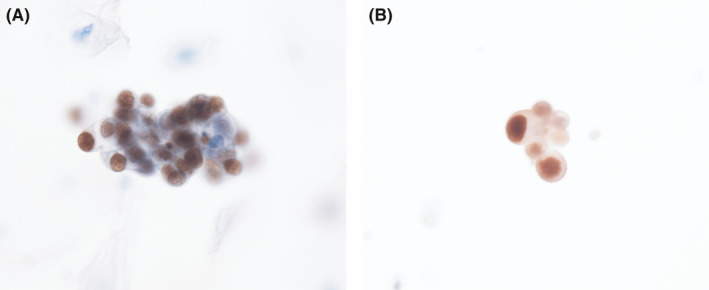
p53 immunocytochemistry. A, RRTCs (Figure 1A) showed positive for p53. B, Large RRTCs in cluster (Figure 1B) expressing p53

RRTCs in all the urine samples (*n *= 68/68, 100%) were positive for p53. The average p53 positivity rate of the RRTCs per sample was 47.7% (range, 3.8%–96.5%; Table 2; Figure 2).

**TABLE 2:** Comparison of p53 immunoexpression between urine cytology and renal biopsy specimens

	Urine cytology	Renal biopsy
p53‐positive RRTCs (*n *= 68)	100% (68/68)	100% (68/68)
p53 positivity rate in RRTCs per sample (mean [range])	47.7% (3.8%–96.5%)	ND
p53/HGD‐positive RRTCs (*n *= 68)	55.9% (38/68)	100% (68/68)
p53/HGD co‐positivity rate in RRTCs per sample (mean [range])	27.6% (4.5%–100%)	ND

Abbreviations: ND, not determined; RRTCs, repair/reactive renal tubular cells.

### 3.5 Correlation between the renal tubular damage rate in renal biopsy and the number and p53 positivity rate of urinary RRTCs

No correlation was observed between the renal tubular damage rate in renal biopsy and the number of urinary RRTCs (Spearman's correlation coefficient = 0.0968; *p* = 0.390). In contrast, there was a weak correlation between renal tubular damage rate and the p53 positivity rate of urinary RRTCs (Spearman's correlation coefficient = 0.2717; *p *= 0.0141).

### 3.6 p53 positivity rate of RRTCs in renal biopsy

Renal biopsy analysis showed that the RRTCs in all the specimens (*n *= 68/68, 100%) in which they appeared based on urine cytology were positive for p53.

### 3.7 p53 and HGD co‐positivity rate of RRTCs in urine cytology

**FIGURE 3:**
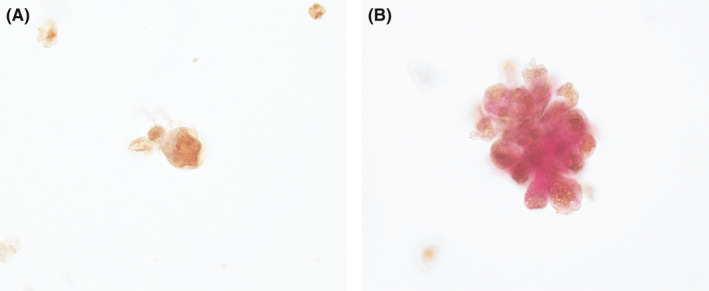
p53 and HGD double immunocytochemistry. A, RRTCs (Figure 1C) demonstrated the expression of only p53. B, RRTCs cluster (Figure 1D) showed the co‐expression of p53 and HGD

Of the 68 urine samples with p53‐positive RRTCs, 38 (55.9%) had RRTCs that were HGD positive for cytoplasm. The average co‐positivity rate of p53 and HGD in the same RRTCs per sample was 27.6% (range, 4.5%–100%; Figure 3). However, urothelial cells and squamous cells were negative for HGD.

### 3.8 p53 and HGD co‐positivity rate of RRTCs in renal biopsy

**FIGURE 4:**
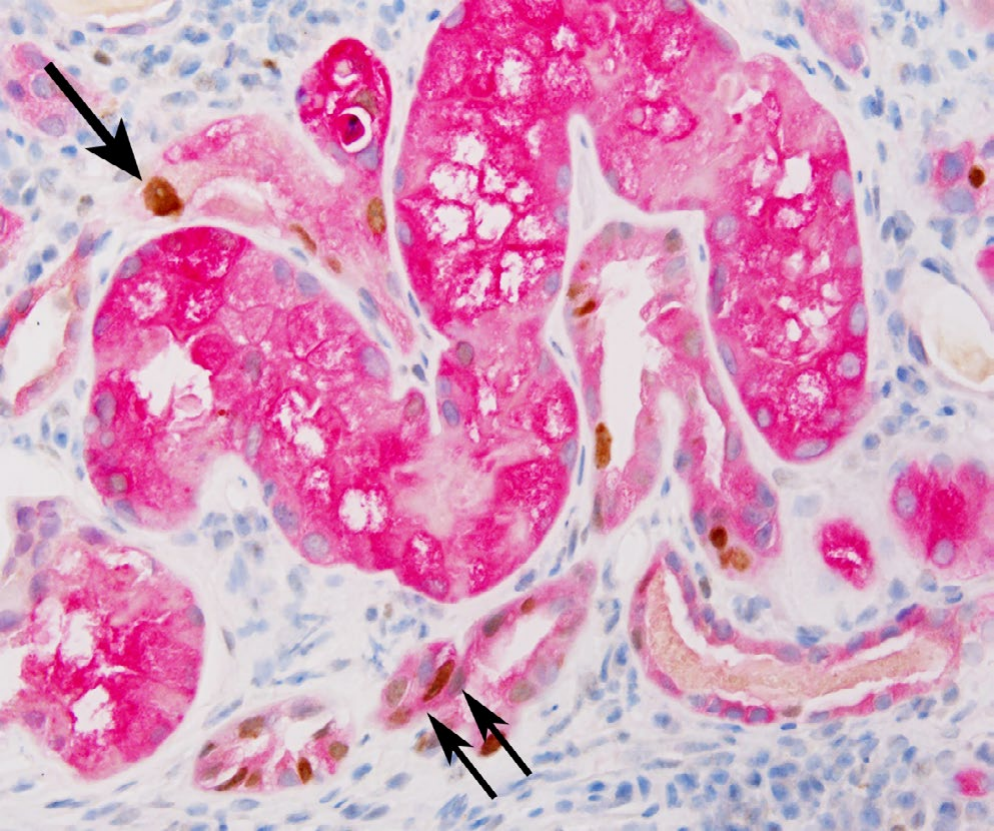
p53 and HGD double immunohistochemistry in renal biopsy. A mixture of p53 only expressed RRTCs (↑) and p53 and HGD co‐expressed RRTCs (↑↑) is shown

Although all renal biopsy specimens detected p53/HGD‐positive RRTCs (*n* = 68/68, 100%), RRTCs expressing only p53 were also observed (Figure 4).

## 4 DISCUSSION

This study revealed that RRTCs appear at a high rate (*n* = 68/80, 85.0%) in the urine of patients with glomerular disease. In our previous study, the appearance rate of RRTCs in urine from glomerular disease patients was 62.5% (*n *= 25/40).^14^ This discrepancy in rates can be attributed to the differences in the experimental methods employed. We used the conventional method for urine cytology in our previous study, whereas we used a modified SurePath method, which has a high cell recovery rate, in this study. Additionally, in this study, the RRTC nuclei were darker than those observed in our previous study,^16^ which may be attributed to the three‐dimensional structure of cells on SurePath slides.^20^ RRTCs exhibit cytomorphological atypia similar to those observed in UCCs and adenocarcinoma cells, including nuclear enlargement, a high N/C ratio, hyperchromasia, nuclear border irregularity, nucleolar prominence, and cannibalism.^13^, ^14^, ^15^ In this study, urinary RRTC cases were experimentally categorized according to the Paris system, and 72.1% of RRTC cases were categorized as atypical urothelial cells, suspicious for HGUC, HGUC, or adenocarcinoma. This result highlights the need for proper distinction RRTCs from UCCs and adenocarcinoma.

In the present study, p53‐positive RRTCs were detected in all urine samples in which RRTCs appeared (*n* = 68); similarly, p53‐positive RRTCs were recognized in all 68 renal biopsy specimens. p53 function is controlled by negative regulators, such as MDM2, and these regulators induce p53 degradation and prevent its accumulation in the nucleus of normal cells.^21^ Consequently, wild‐type p53 in normal cells is rarely detected in immunostaining. However, the interaction between p53 and negative regulators does not function when the cells detect intracellular events, such as DNA damage and oxidative stress. Studies have shown that p53 phosphorylation by various stimuli and stresses leads to stabilization, accumulation, and activation of p53 in the nucleus.^4^, ^21^, ^22^ Through immunohistochemistry, Shimizu et al.^23^ found that p53 was expressed in the renal tubular cells of rats with chronic renal failure. They estimated that reactive oxygen species promote the activation and accumulation of p53 through phosphorylation, thereby suppressing the proliferation of renal tubular cells.^24^ In addition, increased p53 protein levels in renal tubular cells have been reported in ischemic and cisplatin‐induced acute kidney injury mice models and in renal biopsies of patients with acute tubular necrosis.^17^, ^25^, ^26^ We hypothesized that p53‐positive RRTCs will be detected in urine cytologic and renal biopsy specimens in the present study using glomerular disease as a model for reasons similar to those presented in the previous studies. Additionally, this study showed a correlation between the renal tubular damage rate and the p53 positivity rate of urinary RRTCs. Therefore, p53‐positive RRTCs are likely to appear in the urine of patients with glomerular disease and acute tubular necrosis.

Research has described the utility of molecular biology in detecting *TP53* mutation using urine samples, but its methods are time consuming and costly.^27^, ^28^ In contrast, because additional p53 immunostaining can be easily performed as a routine test, p53 immunostaining has been generally used in histopathology and urine cytology as a surrogate marker for *TP53* mutational status in UCCs.^4^, ^5^, ^8^ Previous studies using urine cytology reported that p53 immunocytochemistry could help detect UCCs as well as improve the sensitivity and specificity of the detection.^10^, ^11^, ^12^ In addition, Piaton et al.^9^ indicated that p53 immunocytochemistry might help to identify high‐risk cases of recurrence and progression. However, the previous studies did not consider RRTCs in urine cytologic specimens from patients with glomerular disease. Moreover, these studies used the p53 positivity cutoff values of >5 and >10 positive cells for cell count and >5% for positivity rate.^10^, ^11^, ^12^ In this study, the RRTCs were detected at a mean count of 141.1 cells per sample. They had an average p53 positivity rate of 47.7%, in most cases, which exceeds the corresponding cutoff values defined by previous studies.^10^, ^11^, ^12^ Further studies on RRTCs are needed to establish and standardize the cutoff values for p53 positivity in urine cytology.

RRTCs are positive for vimentin, and vimentin immunocytochemistry has been found to be effective as an objective differentiation method for UCCs.^13^, ^14^, ^15^, ^29^ Vimentin expression in RRTCs results from epithelial‐to‐mesenchymal transition (EMT).^30^, ^31^ EMT is a phenotypic process that involves epithelial cells undergoing multiple changes to assume a mesenchymal cell phenotype. EMT is divided into three different subtypes based on the biological content. Type 1 EMT is associated with implantation, embryo formation, and organ development, whereas type 2 and type 3 EMT can be activated in association with tissue repair and pathological stresses, including various types of inflammation and high‐grade carcinomas.^30^ Type 2 EMT in RRTCs is observed in inflammation and fibrosis, and it can eventually lead to the transformation of epithelial cells into myofibroblasts if the primary inflammation is not removed or attenuated. In contrast, type 3 EMT involves the transformation of epithelial cells into the mesenchymal phenotype of cancer cells to obtain migratory ability at the invasive tumor front.^30^ As a result, type 3 EMT cells are generally limited to the basal layer of the tumor. In voided urine cytology, cells on the slide have been derived from the surface of the epithelium and tumor. This explains why vimentin‐positive cancer cells from low‐ and high‐grade urothelial carcinoma cases were not detected in previously analyzed voided urine cytologic specimens.^15^, ^31^ However, of note, cancer cells from the basal layer may appear in catheterized urine and urine collected after transurethral resection due to the exposure of the basal layer.

Cytologists often focus on only detecting cancer cells; however, urologists are typically motivated to determine the cause of hematuria. As a result, urine samples from patients with glomerulonephritis account for a substantial percentage of urine cytology examinations.^29^ In this study, RRTCs were detected at a high rate in the urine cytologic specimens from patients with glomerular disease. Although cytologists are presently not well versed in the existence and cytologic features of RRTCs, these cells cytomorphologically mimic cancer cells. This study also revealed that RRTCs are positive for p53 immunocytochemistry. Our results suggest that the appearance of RRTCs leads to false positives in urine cytology. Therefore, not only p53 immunostaining but also double immunostaining with p53 and vimentin or HGD should be considered while detecting cancer cells in urine cytology.

In conclusion, we found that RRTCs are positive for p53, with an average positivity rate of RRTCs per sample being 47.7%. Therefore, cytologists must factor in the fact that RRTCs from glomerular disease patients are also positive for p53 to avoid false positives.

## CONFLICT OF INTEREST

The authors have no conflict of interest to declare.

## AUTHOR CONTRIBUTIONS

Methodology: Ohsaki H. Formal analysis: Enomoto K., Nakamura A., Ibuki E., and Ohsaki H. Resources: Matsunaga T., Sofue T., Haba R., and Ohsaki H. Writing‐original draft: Enomoto K. and Ohsaki H. Writing‐review and editing: Hirakawa E., Kamoshida S., and Ohsaki H. Supervision: Ohsaki H.

## ETHICAL APPROVAL

This study was approved by the ethics committees of the Kobe University Graduate School of Health Sciences (approval no 954) and Kagawa University Hospital (approval no H26‐111), and written informed consent was obtained from all patients. It was conducted in accordance with the principles of the Declaration of Helsinki.

## Data Availability

The data that support the findings of this study are available from the corresponding author, [HO], upon reasonable request.
